# Establishing a Urine-Based Biomarker Assay for Prostate Cancer Risk Stratification

**DOI:** 10.3389/fcell.2020.597961

**Published:** 2020-12-10

**Authors:** Jinan Guo, Dale Liu, Xuhui Zhang, Heather Johnson, Xiaoyan Feng, Heqiu Zhang, Alan H. B. Wu, Lingwu Chen, Jiequn Fang, Zhangang Xiao, Kefeng Xiao, Jenny L. Persson, Chang Zou

**Affiliations:** ^1^Shenzhen People’s Hospital, The Second Clinical Medical College, Jinan University, Shenzhen, China; ^2^The First Affiliated Hospital, Southern University of Science and Technology, Shenzhen, China; ^3^Shenzhen Public Service Platform on Tumor Precision Medicine and Molecular Diagnosis, Shenzhen, China; ^4^Department of Bio-Diagnosis, Institute of Basic Medical Sciences, Beijing, China; ^5^Olympia Diagnostics, Inc., Sunnyvale, CA, United States; ^6^Clinical Laboratories, San Francisco General Hospital, San Francisco, CA, United States; ^7^Department of Urology, The First Affiliated Hospital, Sun Yat-sen University, Guangzhou, China; ^8^Laboratory of Molecular Pharmacology, Department of Pharmacology, School of Pharmacy, Southwest Medical University, Luzhou, China; ^9^Department of Molecular Biology, Umeå University, Umeå, Sweden; ^10^Division of Experimental Cancer Research, Department of Translational Medicine, Lund University, Malmö, Sweden

**Keywords:** urine-based biomarker, Gene Panel, prostate cancer, Gleason score, cancer risk stratification

## Abstract

One of the major features of prostate cancer (PCa) is its heterogeneity, which often leads to uncertainty in cancer diagnostics and unnecessary biopsies as well as overtreatment of the disease. Novel non-invasive tests using multiple biomarkers that can identify clinically high-risk cancer patients for immediate treatment and monitor patients with low-risk cancer for active surveillance are urgently needed to improve treatment decision and cancer management. In this study, we identified 14 promising biomarkers associated with PCa and tested the performance of these biomarkers on tissue specimens and pre-biopsy urinary sediments. These biomarkers showed differential gene expression in higher- and lower-risk PCa. The 14-Gene Panel urine test (*PMP22*, *GOLM1*, *LMTK2*, *EZH2*, *GSTP1*, *PCA3*, *VEGFA*, *CST3*, *PTEN*, *PIP5K1A*, *CDK1*, *TMPRSS2*, *ANXA3*, and *CCND1*) was assessed in two independent prospective and retrospective urine study cohorts and showed high diagnostic accuracy to identify higher-risk PCa patients with the need for treatment and lower-risk patients for surveillance. The AUC was 0.897 (95% CI 0.939–0.855) in the prospective cohort (*n* = 202), and AUC was 0.899 (95% CI 0.964–0.834) in the retrospective cohort (*n* = 97). In contrast, serum PSA and Gleason score had much lower accuracy in the same 202 patient cohorts [AUC was 0.821 (95% CI 0.879–0.763) for PSA and 0.860 (95% CI 0.910–0.810) for Gleason score]. In addition, the 14-Gene Panel was more accurate at risk stratification in a subgroup of patients with Gleason scores 6 and 7 in the prospective cohort (*n* = 132) with AUC of 0.923 (95% CI 0.968–0.878) than PSA [AUC of 0.773 (95% CI 0.852–0.794)] and Gleason score [AUC of 0.776 (95% CI 0.854–0.698)]. Furthermore, the 14-Gene Panel was found to be able to accurately distinguish PCa from benign prostate with AUC of 0.854 (95% CI 0.892–0.816) in a prospective urine study cohort (*n* = 393), while PSA had lower accuracy with AUC of 0.652 (95% CI 0.706–0.598). Taken together, the 14-Gene Panel urine test represents a promising non-invasive tool for detection of higher-risk PCa to aid treatment decision and lower-risk PCa for active surveillance.

## Introduction

Prostate cancer (PCa) is the third most prevalent cancer with 191,930 estimated new cases in 2020 that represents 10.6% of all new cancer cases in the United States (National Cancer Institute^1^, date of last access: 10/29/2020). Some studies have shown that 90% of low-risk patients managed to survive over 10 years with active surveillance (Tosoian et al., 2015). To avoid overtreatment and reduce potential treatment side effects such as urinary and erectile dysfunctions, active surveillance can be used for patients with low-risk PCa. Although there is no absolute consensus on the criteria of high-risk and low-risk PCa, Gleason score, cancer stage, percent of biopsy core with cancer, and PSA (prostate-specific antigen) have been used in clinical practice and many studies for PCa risk stratification, sometimes with additional consideration such as PSA density (Selvadurai et al., 2013; Klotz et al., 2015; Tosoian et al., 2016; Jones et al., 2018). However, Gleason score, cancer stage, and cancer core information are all obtained from biopsy, and frequent or periodic biopsies are not amenable for patients. There is an urgent need to develop a non-invasive and convenient test for accurate PCa risk stratification for active surveillance and treatment decision.

Measurement of the expression level of one or more PCa-specific biomarkers in urine samples has been used for PCa diagnosis and is a more convenient and non-invasive method of liquid biopsy (Groskopf et al., 2006; Guo et al., 2018). PCa is a heterogeneous cancer, and mutation and alteration of various genes contribute to cancer progression, recurrence, and metastasis. Currently, there is no clinical parameter or a single biomarker that is sufficient to provide accurate PCa risk stratification and prognosis. Urine has emerged as a viable source for non-invasive detection of PCa, as the urine can be easily obtained. More importantly, prostate tumors actively release cancer cells into the urine, despite only a small amount of cancer cells present in the urine. Thus, only the markedly altered cancer-specific biomarkers may be detected in urine samples (Groskopf et al., 2006; Guo et al., 2018).

Previously, we identified an 8-Gene Panel, which consists of *PMP22* (peripheral myelin protein 22), *GOLM1* (Golgi membrane protein 1), *LMTK2* (Lemur tyrosine kinase 2), *EZH2* (enhancer of zeste homolog 2), *GSTP1* (glutathione *S*-transferase Pi 1), *PCA3* (prostate cancer antigen 3), *HPN* (hepsin), and *FN1* (fibronectin) and found that it was able to distinguish Gleason score >6 and Gleason score ≤6 PCa with good sensitivity and specificity using prostate specimens (Xiao et al., 2016). In addition, we identified more genes with differential expression in the prostate specimen cohort (data not published). Among them, several genes have been identified as PCa therapeutic targets including *VEGFA* (vascular endothelial growth factor A), *PTEN* (phosphatase and tensin homolog), *PIP5K1A* (phosphatidylinositol-4-phosphate 5-kinase type 1 alpha), and *CDK1* (cyclin-dependent kinase 1) (Semenas et al., 2014; Koryakina et al., 2015; Sarwar et al., 2016; Mateo et al., 2017; Cereda et al., 2018). *CST3* (cystatin C) was found to have differential expression in aggressive and indolent PCa (Jiborn et al., 2006). *TMPRSS2* (transmembrane protease, serine 2) is a target gene of AR, and its fusion with *ERG* occurs in 40–80% of PCa (Yu et al., 2010; Ito et al., 2018). *ANXA3* (calcium-dependent phospholipid-binding protein Annexin A3) has been detected in PCa urine samples, and the decreased *ANXA3* expression was negatively correlated with PCa development (Wu N. et al., 2013; Jeun et al., 2017). *CCND1* (cyclin D1) has been shown to play an important role in androgen-enhanced DNA damage repair, and androgen-mediated recruitment of cyclin D1 to DNA repair sites may contribute to PCa cells’ resistance to treatment for DNA damage (Casimiro et al., 2016).

In this study, we aimed to develop a combination of PCa-specific biomarkers, which are associated with the complexity and heterogeneous nature of PCa and establish a urine-based test by using the identified biomarker combination to detect higher-risk PCa for treatment and lower-risk PCa for active surveillance. We showed that a 14-Gene Panel using urine samples collected before prostate biopsy or radical prostatectomy may act as a promising novel tool to guide decision making for PCa treatment and management.

## Materials and Methods

### PCa Tissue Specimen Cohort

A PCa dataset cohort with clinicopathological information of patients was obtained from The Cancer Genome Atlas (TCGA) Prostate Adenocarcinoma Provisional dataset consisting of tumors taken from primary site (*n* = 495) as described (Cancer Genome Atlas Research Network, 2015). The dataset contains quantitative mRNA expression Z-Score by RNA Seq V2 RSEM analysis using 333 unique prostate tissue specimens from radical prostatectomy of patients diagnosed with PCa. Among them, 290 specimens have viable gene expression data and were used for our study. The quantitative mRNA expression data of genes was obtained from the database.

### Prospective and Retrospective Urine Cohorts

The urine sample cohort comprising of 393 patients was obtained from a prospective study approved by the Institutional Review Board of Shenzhen People’s Hospital, Shenzhen, China (Study Number: P2014-006) (referred as prospective urine cohort). The urine samples were collected from seven hospitals collaborated in the study before needle biopsy, radical prostatectomy, or electro-prostatectomy from patients with informed consent. Ninety-seven samples in a retrospective cohort were randomly picked from archived samples at the Cooperative Human Tissue Network (CHTN) Southern Division with IRB approval from San Francisco General Hospital, San Francisco, United States (IRB #: 15-15816) and prior patient consent (referred as retrospective PCa urine cohort). PCa diagnosis and Gleason scores were based on pathological analysis of prostate specimens from biopsy, radical prostatectomy, or electro-prostatectomy with consistent procedures and recorded on pathology reports. Among the prospective cohort patients, 202 patients were diagnosed to have PCa (referred as prospective PCa urine cohort), while 191 had benign prostate (benign prostatic hyperplasia or prostatitis). The pathological diagnosis of PCa risk was defined based on the National Comprehensive Cancer Network (NCCN) guidelines. NCCN recommends patients with very high, high, and unfavorable intermediate risk to receive immediate treatment, while most patients with very low, low, and favorable intermediate risk are suggested to be placed on active surveillance. Therefore, in this study, we classified patients with very high, high, and unfavorable intermediate risk as higher-risk PCa who need treatment, and patients with very low, low, and favorable intermediate risk to be lower risk patients who need active surveillance. Such classification to separate the cancer patients into two groups with and without the need of immediate treatment is clinically relevant and can help in treatment decision making in clinical practice. Based on NCCN guidelines, the higher-risk PCa patients in our study were classified as meeting any of the following criteria: Gleason score >7, Gleason score 4 + 3 = 7, cancer stage ≥T3, PSA >20 ng/ml at diagnosis, and more than half of the biopsy core with cancer. The rest of the patients were classified as lower-risk PCa. In the prospective PCa urine cohort, 149 patients had higher risk PCa, while 53 had lower-risk PCa. Also, in the retrospective PCa urine cohort, 47 patients had higher-risk PCa, while 50 had lower-risk PCa.

### Urine Sample Collection

For prospective urine samples, a volume of 15–45 ml urine was voided into a 50-ml urine collection tube containing DNA/RNA preservative AssayAssure (Thermo Fisher Scientific, Waltham, MA, United States). The urine pellets obtained after centrifugation at 1,000 × *g* for 10 min were washed with phosphate-buffered saline followed by a second centrifugation at 1,000 × *g* for 10 min. The cell pellet was processed for RNA purification or immediately frozen on dry ice and stored at −80°C until future purification. All samples were de-identified and coded with patient numbers to ensure the privacy of the donors in accordance with the Health Insurance Portability and Accountability Act (HIPAA). For retrospective urine study samples, a volume of 15-ml urine sample was collected before pathological diagnosis of prostate specimens from needle biopsy or radical prostatectomy. The urine pellet obtained after centrifugation was immediately flash frozen and stored at −80°C. All samples were de-identified and coded with patient numbers to ensure the privacy of the donors in accordance with the Health Insurance Portability and Accountability Act (HIPAA).

### Quantitative Measurement of Gene Expression of 14 Biomarkers

The urine pellet was thawed at 37°C and washed with cold phosphate-buffered saline followed by centrifugation at 1,000 × *g* for 10 min. Total RNA was purified from the cell pellet using Quick-RNA MicroPrep Kit (Zymo Research, Irvine, CA, United States) following the manufacturer’s protocol. cDNA was generated by reverse transcription of 100 ng purified RNA using either iScript Reverse Transcription Supermix for qRT-PCR (Bio-Rad, Hercules, CA, United States) or High Capacity cDNA Reverse Transcription kit (Life Technologies, Foster City, CA, United States) following the manufacturers’ directions. cDNA was then preamplified using preamplification mixture (Olympia Diagnostics, Sunnyvale, CA, United States) following the protocol (Johnson et al., 2020). Real-time qRT-PCR was performed to assess expression levels of the 14 genes. The primers and probes of the biomarker genes were predesigned assays purchased from Integrated DNA Technologies (San Diego, CA, United States). Real-time qRT-PCR was performed on ABI Quantstudio 6 Real-Time PCR System (Thermo Fisher, Foster City, CA, United States). The PCR reaction was set in 10-μl volume consisting of cDNA transcribed and preamplified from 0.2 ng of purified RNA, 5 μl of 2 × TaqMan^®^ Universal PCR Master Mix (Life Technologies, Foster City, CA, United States), 500 nM each of forward and reverse amplification primer, and a 250-nM probe. The real-time PCR cycling condition was set as the following: 95°C for 10 min for polymerase activation, followed by 40 cycles of 95°C for 15 s and 60°C for 1 min.

### Data Analysis and Diagnosis of the 14-Gene Panel by Algorithms

All tests were performed blindly without prior knowledge of patient information. The data analysis was performed using ABI Quantstudio 6 software (Life Technologies, Foster City, CA, United States). The level of a housekeeping gene beta-actin mRNA was measured in each sample for gene expression normalization to control variations of cDNA quantity in the patient samples. The cycle threshold (*C*t) value of each gene in the 14-Gene Panel was divided by the Ct value of the beta-actin mRNA as the normalized mRNA expression value of the gene [Ct_*S*_ = Ct (sample)/Ct (actin)]. For each gene, triplicate PCRs were performed to average the *C*t values.

For PCa risk stratification, the CtS values of the 14 genes in the panel were used to generate a classification score (Stratification D Score) for each urine sample using the following stratification algorithm:

$$C_{\mathrm{HigherRisk}}=A_{H}+{\mathrm{CtS}}_{1}*H_{1}+{\mathrm{CtS}}_{2}*H_{2\,\ldots }+{\mathrm{CtS}}_{14}*H_{14}$$

$$+{\mathrm{CtS}}_{1}*{\mathrm{CtS}}_{1}*H_{1*1}+{\mathrm{CtS}}_{1}*{\mathrm{CtS}}_{2}*H_{1*2\,\ldots }$$

$$+CtS_{1}*CtS_{14}*H_{1*14}+{\mathrm{CtS}}_{2}*{\mathrm{CtS}}_{2}*H_{2*2\,\ldots }$$

$$+CtS_{2}*CtS_{25}*H_{2*14\,\ldots }+CtS_{14}*CtS_{14}*H_{14*14}$$

$$C_{\mathrm{LowerRisk}}=B_{L}+{\mathrm{CtS}}_{1}*L_{1}+{\mathrm{CtS}}_{2}*L_{2\,\ldots }+{\mathrm{CtS}}_{14}*L_{14}$$

$$+CtS_{1}*{\mathrm{CtS}}_{1}*L_{1*1}+{\mathrm{CtS}}_{1}*{\mathrm{CtS}}_{2}*L_{1*2\,\ldots }$$

$$+CtS_{1}*CtS_{14}*L_{1*14}+CtS_{2}*CtS_{2}*L_{2*2\,\ldots }$$

$$+CtS_{2}*CtS_{14}*L_{2*14\,\ldots }+CtS_{14}*CtS_{14}*L_{14*14}$$

Stratification *D* Score = *C*_HigherRisk_ − *C*_LowerRisk_

where *A*_*H*_ is the higher-risk PCa constant, *B*_*L*_ is the lower-risk PCa constant, CtS_1_ through CtS_14_ are the CtS values of gene 1 through gene 14, *H*_1_ through *H*_14_ are higher-risk PCa regression coefficients of gene 1 through gene 14, *H*_1^*1_ through *H*_14^*14_ are the gene 1 and gene 1 cross higher-risk PCa regression coefficients through gene 14 and gene 14 cross higher-risk PCa regression coefficients, *L*_1_ through *L*_14_ are the lower-risk PCa regression coefficients of gene 1 through gene 14, and *L*_1^*1_ through *L*_14^*14_ are the gene 1 and gene 1 cross lower-risk PCa regression coefficients through gene 14 and gene 14 cross lower-risk PCa regression coefficients. The sample was diagnosed to be higher-risk PCa when the Stratification *D* Score was >0, whereas the sample was diagnosed to be lower-risk PCa when the stratification *D* Score was ≤0.

For PCa diagnosis, CtS values of the 14 genes in the panel were used to generate a classification score (Diagnosis *D* Score) for each sample using the diagnosis algorithm as described (Johnson et al., 2020).

### Statistical Analysis

A statistical analysis software program XLSTAT (Addinsoft, New York, NY, United States) was used to analyze the data. In the biomarker validation study, the box plot analysis and two sample *t*-tests were performed on each gene in the panel to assess the differential gene expression in higher- and lower-risk PCa and statistical significance *p*-value by using XLSTAT. The discriminating score (F1 score) was calculated by discriminant analysis (DA) using XLSTAT and was used in box plot of the 14-Gene Panel. The AUC of ROC curve and the corresponding 95% confidence intervals (CI) were calculated using DA. For risk stratification, the diagnosis of each sample by the risk stratification algorithm was compared to their pathological diagnosis of higher or lower risk to assess diagnostic performance values including sensitivity, specificity, positive predictive value (PPV), and negative predictive value (NPV), and their respective 95% CI using DA. For cancer diagnosis, the diagnosis of each sample by the diagnosis algorithm was compared to pathological diagnosis to assess the diagnostic performance of the 14-Gene Panel. The diagnostic performance of PSA in the prospective urine cohort was assessed by DA.

## Results

### Identification of 14-Gene Panel With 290 PCa Specimen Cohort

**FIGURE 1:**
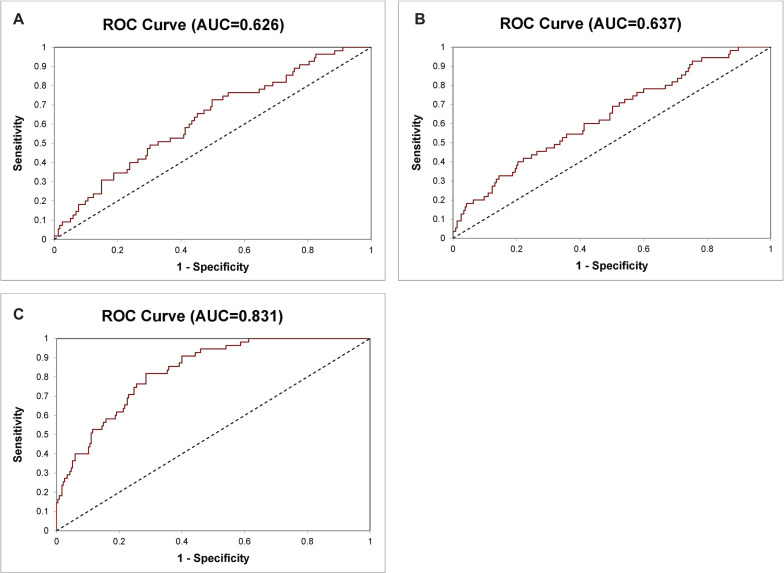
Receiver operating characteristic (ROC) curve for diagnosis of Gleason score >6 and Gleason score ≤6 prostate cancer (PCa) in the 290 prostate specimen cohort. **(A)** The 8-Gene Panel, **(B)** the 6-Gene Panel, **(C)** the 14-Gene Panel.

In our previous study, we have shown that an 8-Gene Panel, which consists of *PMP22*, *GOLM1*, *LMTK2*, *EZH2*, *GSTP1*, *PCA3*, *HPN*, and *FN1*, can be used as a predictive model to distinguish Gleason score >6 PCa from Gleason score ≤6 PCa with high sensitivity and specificity in cancer tissues from two PCa patient cohorts (*n* = 87 and *n* = 158) (Xiao et al., 2016). In this study, we examined the predictive accuracy of the 8-Gene Panel in a larger PCa patient cohort from TCGA dataset (*n* = 290), on the basis of their mRNA expression profiles in cancer tissues. The patients’ characteristics of the cohort are shown in Table 1. This 8-Gene Panel model was used for evaluating the model discrimination using ROC analysis. The diagnostic accuracy for discriminating Gleason score >6 from Gleason score ≤6 PCa was relatively low as shown by the AUC of 0.626 (95% CI 0.703–0.549) (Figure 1A). The sensitivity was 98.30% (95% CI 99.95–90.64%), specificity was 5.45% (95% CI 11.46–0.55%), positive predictive value (PPV) was 81.63% (95% CI 86.14–77.11%), and negative predictive value (NPV) was 42.90% (95% CI 79.52–6.20%) (*p* < 0.001) (Table 2).

**TABLE 1:** Characteristics of patients.

	**Prostate specimen cohort**	**Prospective PCa urine cohort**	**Retrospective PCa urine cohort**	**Prospective urine cohort**
Number of patients	290	202	97	393
Number of cancer	290	202	97	202
Number of benign prostate	0	0	0	191
Mean diagnosis age	61	69	62	69
**Gleason score (%)**				
Group 1: ≤6	55 (18.97%)	52 (25.74%)	32 (32.99%)	52 (25.74%)
Group 2: 7 (3 + 4)	89 (30.69%)	42 (20.79%)	23 (23.71%)	42 (20.79%)
Group 3: 7 (4 + 3)	68 (23.45%)	41 (20.30%)	25 (25.77%)	41 (20.30%)
Group 4: 8	39 (13.45%)	36 (17.82%)	5 (5.15%)	36 (17.82%)
Group 5: 9 or 10	39 (13.45%)	31 (15.35%)	4 (4.12%)	31 (15.35%)
Unknown	0	0	8 (8.25%)	0
PSA < 10 ng/dl (%)	112 (38.62%)	61 (30.19%)	NA	185 (47.07%)
PSA 10–20 ng/dl (%)	29 (10.00%)	42 (20.79%)	NA	92 (23.41%)
PSA > 20 ng/dl (%)	21 (7.24%)	96 (47.52%)	NA	111 (28.24%)
Unknown	128 (44.14%)	3 (1.49%)	NA	5 (1.27%)
Metastasis (%)	NA	47 (23.27%)	4 (4.12%)	47 (11.96%)

**TABLE 2:** Sensitivity, specificity, positive predictive value (PPV), and negative predictive value (NPV) of 8-Gene-Panel and 14-Gene Panel for diagnosis of Gleason Score >6 and Gleason Score ≤ 6 PCa in a 290 prostate specimen cohort.

	**8-gene panel**			**14-gene panel**		
	**Positive**	**Negative**	**Total**	**Positive**	**Negative**	**Total**
Gleason score >6 PCa	231	4	235	190	45	235
Gleason score ≤6 PCa	52	3	55	21	34	55
Sensitivity (95% CI)	98.30 (99.97–96.63)%			80.85 (86.45–75.26)%		
Specificity (95% CI)	5.45 (31.15–20.24)%			61.82 (78.15–45.49)%		
PPV (95% CI)	81.63 (86.62–76.63)%			90.05 (94.30 85.79)%		
NPV (95% CI)	42.86 (98.86–13.14)%			43.04 (59.68–26.39)%		

Since the six biomarkers *PMP22*, *GOLM1*, *LMTK2*, *EZH2*, *GSTP1*, and *PCA3* in this 8-Gene Panel exhibited most differential gene expression pattern between Gleason score >6 PCa and Gleason score ≤6 PCa in cancer tissues from TCGA patient cohort and our prostate tissue cohorts previously used, we found that a combination of these six genes as a 6-Gene Panel model improved the ability to distinguish the two groups of PCa with sensitivity of 94.47% (95% CI 97.39–91.55%), specificity of 18.18% (95% CI 28.38–7.99%), PPV of 83.15% (95% CI 87.64–78.66%), and NPV of 43.48% (95% CI 63.74–23.22%) (*p* < 0.001). The ROC curve analysis was performed, and the result showed an AUC of 0.637 (95% CI 0.713–0.561) (Figure 1B).

We wanted to improve the 6-Gene Panel model by including additional PCa-specific biomarkers to the combination. We added genes including *VEGFA*, *CST3*, *PTEN*, *PIP5K1A*, *CDK1*, *TMPRSS2*, *ANXA3*, and *CCND1* based on their roles and their differential gene expression levels in cancer tissues between Gleason score >6 and Gleason score ≤6 PCa. The 14-Gene Panel was tested in the 290 tissue cohort. The ROC curve analysis was performed, and the AUC was 0.831 (95% CI 0.881–0.781) (Figure 1C), which was much higher than that of the 6-Gene Panel (AUC was 0.637). The 14-Gene Panel model showed sensitivity of 80.85% (95% CI 85.88–75.82%), specificity of 61.82% (95% CI 74.66–48.98%), PPV of 90.05% (95% CI 94.09–86.01%), and NPV of 43.04% (95% CI 53.96–32.12%) (*p* < 0.0001) (Table 2).

### Validation of Differential Gene Expression of the 14 Genes in a Prospective PCa Urine Cohort

Using conventional clinicopathological parameters such as Gleason score, cancer stage, biopsy core with cancer, and pre-operative PSA for PCa stratification and surveillance has limitations. The pathological measures, such as Gleason score, cancer stage, and biopsy core with cancer, all depend on tissue biopsy, which are limited by sampling location and assessment errors. In addition, biopsy cannot be performed periodically during cancer surveillance. Further, preoperative PSA cannot accurately distinguish high and low risk PCa. Thus, a non-invasive urine test using PCa-specific biomarkers is independent of biopsy tissue or PSA and may provide more accurate risk assessment for treatment decision and more convenient periodic monitoring for cancer surveillance. Thus, we intended to develop a non-invasive urine test using the 14-Gene Panel to separate the cancer patients into two groups with and without the need for immediate treatment. Based on the NCCN guidelines for PCa diagnosis and treatment, we classified patients with very high, high, and unfavorable intermediate risk as higher-risk PCa since these patients need immediate treatment, and patients with very low, low, and favorable intermediate risk as lower-risk PCa since most of these patients need active surveillance without immediate treatment. Such stratification is clinically relevant and can help in treatment decision making in clinical practice. We named these two groups as higher- and lower-risk PCa to avoid confusion with the NCCN’s terminology of cancer risk groups. We first tested if the 14 biomarkers had differential gene expression in higher- and lower-risk PCa patients. We used prospectively collected urine samples from 202 PCa patients, among which 142 (70.30%) had higher-risk PCa and 60 (29.70%) had lower-risk PCa.

**FIGURE 2:**
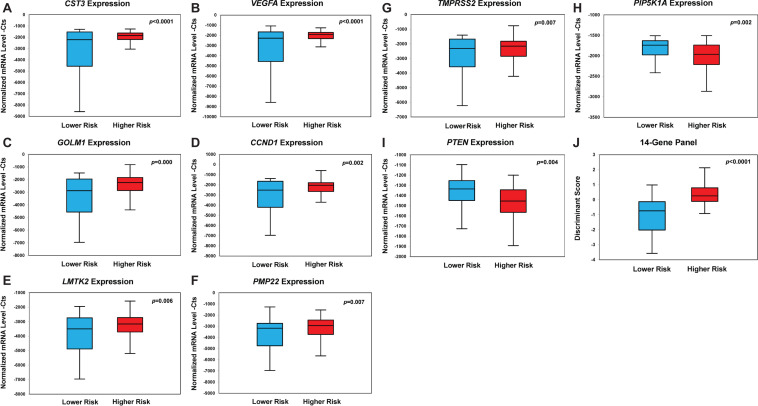
Box plots of genes with statistically significant differential gene expression in the prospective PCa urine cohort (*n* = 202). Box plot of expression of *CST3* **(A)**, *VEGFA* **(B)**, *GOLM1* **(C)**, *CCND1* **(D)**, *LMTK2 (E)*, *PMP22* **(F)**, *TMPRSS2* **(G)**, *PIP5K1A* **(H)**, *PTEN* **(I)**, and the 14-Gene Panel **(J)**. Normalized mRNA level—Cts is defined as cycle threshold (Ct) (sample)/Ct (actin) * 1,000.

The mRNA expression data of each biomarker in urinary sediment RNA samples from 202 patients was quantified by qRT-PCR. Among the 14 biomarkers, we found that mRNA expression of *CST3*, *VEGFA*, *GOLM1*, *CCND1*, *LMTK2*, *PMP22*, and *TMPRSS2* was significantly upregulated in urine samples from higher-risk PCa patients compared with that of lower-risk group (Figures 2A–G). The mean value of relative mRNA expression Ct_*S*_ of these genes all increased significantly in higher-risk PCa compared to lower-risk PCa [*CST3* was increased by 1,008 (*p* < 0.0001), *VEGFA* was increased by 953 (*p* < 0.0001), *GOLM1* was increased by 813 (*p* = 0.000), *CCND1* was increased by 727 (*p* = 0.002), *LMTK2* was increased by 552 (*p* = 0.006), *PMP22* was increased by 534 (*p* = 0.007), and *TMPRSS2* was increased by 523 (*p* = 0.007)].

In contrast, the mRNA expression of *PIP5K1A* and *PTEN* was significantly downregulated in higher-risk PCa compared to lower-risk PCa (Figures 2H,I). The mean value of the relative mRNA expression was significantly decreased in higher-risk PCa compared to lower-risk PCa by 274 for *PIP5K1A* (*p* = 0.002) and 121 for *PTEN* (*p* = 0.004). The mRNA expression of *CDK1*, *EZH2*, *PCA3*, *ANXA3*, and *GSTP1* also exhibited large differential expression pattern between the two risk groups, although the statistical significance was not achieved (Supplementary Figure 1). When the 14 biomarkers were combined, the 14-Gene Panel displayed a striking differential gene expression pattern that can significantly distinguish higher-risk from lower-risk PCa (*p* < 0.0001) (Figure 2J).

### Diagnostic Performance of the 14-Gene Panel in a Prospective PCa Urine Cohort

**FIGURE 3:**
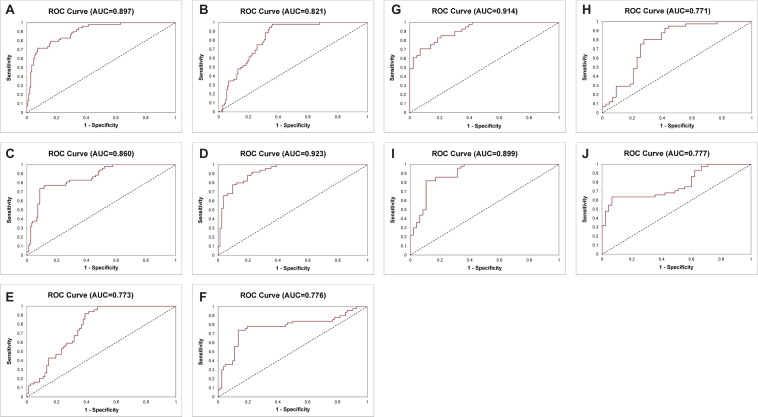
Receiver operating characteristic curves of the 14-Gene Panel, serum prostate-specific antigen (PSA) and Gleason score for diagnosis of higher-risk and lower-risk PCa. ROC curve of the 14-Gene Panel **(A)**, PSA **(B)**, and Gleason score **(C)** in the prospective PCa urine cohort (*n* = 202). ROC curve of the 14-Gene Panel **(D)**, PSA **(E)**, and Gleason score **(F)** in patients with Gleason scores 6 and 7 in the prospective PCa urine cohort (*n* = 132). ROC curve of the 14-Gene Panel **(G)** and PSA **(H)** in patients with Gleason score 7 in the prospective PCa urine cohort (*n* = 84). ROC curve of the 14-Gene Panel **(I)** and Gleason score **(J)** in the retrospective PCa urine cohort (*n* = 97).

Next, the bioinformatics tool was used to evaluate Ct_*S*_ values of mRNA data of the 14-Gene Panel in urinary sediment RNA to generate a stratification algorithm to classify samples as higher- or lower-risk PCa. The diagnostic performance of the 14-Gene Panel was assessed in the 202 patient prospective PCa urine cohort. The ROC analysis was performed, and the diagnostic accuracy was shown by AUC of 0.897 (95% CI 0.939–0.855) (*p* < 0.0001) (Figure 3A). As shown in Table 3, the 14-Gene Panel had sensitivity of 83.22% (95% CI 89.22–77.22%), specificity of 79.25% (95% CI 90.16–68.33%), PPV of 91.85% (95% CI 96.47–87.24%), and NPV of 62.69% (95% CI 74.27–51.11%). In contrast, serum PSA [with AUC of 0.821 (95% CI 0.879–0.763)] and Gleason score [with AUC of 0.860 (95% CI 0.910–0.810)] had lower AUC (Figures 3B,C) than the 14-Gene Panel (with AUC of 0.897). In addition, both PSA and Gleason score had low sensitivity [46.26% (95% CI 54.32–38.20%) for PSA and 43.62% (95% CI 51.59–35.66%) for Gleason score], higher specificity [98.08% (95% CI 101.81–94.34%) for PSA and 98.11% (95% CI 101.78–94.45%) for Gleason score], higher PPV [98.55% (95% CI 101.37–95.73%) for PSA and 98.48% (95% CI 101.43–95.54%) for Gleason score], and lower NPV [39.23% (95% CI 47.62–30.84%) for PSA and 38.24% (95% CI 46.40–30.07%) for Gleason score] (Table 3). This result suggests that the 14-Gene Panel is superior to preoperative PSA and biopsy Gleason score as a more accurate tool for PCa risk stratification.

**TABLE 3:** Sensitivity, specificity, PPV, and NPV of 14-Gene-Panel, prostate-specific antigen (PSA) and Gleason score for diagnosis of higher-risk and lower-risk prostate cancer (PCa) in a prospective PCa urine cohort.

	**14-Gene Panel**			**PSA**			**Gleason Score**		
	**Positive**	**Negative**	**Total**	**Positive**	**Negative**	**Total**	**Positive**	**Negative**	**Total**
Higher risk	124	25	149	68	79	147	65	84	149
Lower risk	11	42	53	1	51	52	1	52	53
Total	135	67	202	69	130	199	66	136	202
Sensitivity (95% CI)	83.22 (89.22–77.22)%			46.26 (54.32–38.20)%			43.62 (51.59–35.66)%		
Specificity (95% CI)	79.25 (90.16–68.33)%			98.08 (101.81–94.34)%			98.11 (101.78–94.45)%		
PPV (95% CI)	91.85 (96.47–87.24)%			98.55 (101.37–95.73)%			98.48 (101.43–95.54)%		
NPV (95% CI)	62.69 (74.27–51.11)%			39.23 (47.62–30.84)%			38.24 (46.40–30.07)%		

### Performance of the 14-Gene Panel to Stratify Cancer Risk in Gleason Scores 6 and 7 Patients

As shown above, the 14-Gene Panel was found to be able to distinguish higher-risk (very high, high, and unfavorable intermediate risk according to the NCCN risk classification) and lower-risk (very low, low, and favorable intermediate risk according to NCCN) patients. In addition, stratification of higher and lower-risk for patients with Gleason scores 6 and 7 may have more clinical relevance as it is especially important and useful to identify higher-risk patients for treatment and lower-risk patients for surveillance in patients with low-grade (Gleason score 6) and intermediate-grade (Gleason score 7) PCa. Thus, we tested the diagnostic performance of the 14-Gene Panel urine test for risk stratification in Gleason scores 6 and 7 patients in the prospective PCa urine cohort (*n* = 132). ROC analysis was performed, and high diagnostic accuracy was shown by an AUC of 0.923 (95% CI 0.968–0.878) in Gleason scores 6 and 7 patients (*p* < 0.0001) (Figure 3D). The 14-Gene Panel had sensitivity of 82.93% (95% CI 91.07–74.78%), specificity of 80.00% (95% CI 91.09–68.91%), PPV of 87.18% (95% CI 94.60–79.76%), and NPV of 74.07% (95% CI 85.76–62.39%) (Table 4). As a comparison, the diagnostic performance of serum PSA and Gleason score in Gleason scores 6 and 7 patients were also assessed. The result showed low AUC of 0.773 (95% CI 0.852–0.694) for PSA and 0.776 (95% CI 0.854–0.698) for Gleason score (Figures 3E,F), sensitivity of 42.68% (95% CI 53.39–31.98%) for PSA and 86.59% (95% CI 93.76–79.21%) for Gleason score, specificity of 100% (95% CI 100–100%) for PSA and 74.00% (95% CI 86.16–61.84%) for Gleason score, PPV of 100% (95% CI 100–100%) for PSA and 84.52% (95% CI 92.26–76.79%) for Gleason score, and NPV of 51.04% (95% CI 61.04–41.04%) for PSA and 77.08% (95% CI 88.97–65.19%) for Gleason score (Table 4). This suggests that the 14-Gene Panel is more accurate at PCa risk stratification in the Gleason scores 6 and 7 patient population than PSA and Gleason score.

**TABLE 4:** Sensitivity, specificity, PPV, and NPV of 14-Gene-Panel, PSA, and Gleason score for diagnosis of higher-risk and lower-risk PCa in Gleason scores 6 and 7 patients in a prospective PCa urine cohort.

	**14-gene panel**			**PSA**			**Gleason score**		
	**Positive**	**Negative**	**Total**	**Positive**	**Negative**	**Total**	**Positive**	**Negative**	**Total**
Higher risk	68	14	82	35	47	82	71	11	82
Lower risk	10	40	50	0	49	49	13	37	50
Total	78	54	132	35	96	131	84	48	132
Sensitivity (95% CI)	82.93 (91.07–74.78)%			42.68 (53.39–31.98)%			86.59 (93.96–79.21)%		
Specificity (95% CI)	80.00 (91.09–68.91)%			100 (100–100)%			74.00 (86.16–61.84)%		
PPV (95% CI)	87.18 (94.60–79.76)%			100 (100–100)%			84.52 (92.26–76.79)%		
NPV (95% CI)	74.07 (85.76–62.39)%			51.04 (61.04–41.04)%			77.08 (88.97–65.19)%		

### Assessment of the 14-Gene Panel to Distinguish Gleason Score 7 Subgroups

For Gleason score 7 patients, it is clinically useful to distinguish between 4 + 3 = 7 and 3 + 4 = 7 patients since they have different clinical outcome and NCCN recommends most patients with Gleason score 4 + 3 = 7 to take immediate treatment, while the choice of treatment or surveillance in patients with Gleason score 3 + 4 = 7 depends on other factors such as cancer stage, PSA, and biopsy core. Therefore, we tested the ability of the 14-Gene Panel urine test to distinguish Gleason score 4 + 3 = 7 from 3 + 4 = 7 patients in the prospective PCa urine cohort (*n* = 84). ROC analysis was performed, and good diagnostic accuracy was shown by AUC of 0.914 (95% CI 0.977–0.851) (*p* < 0.0001) (Figure 3G). The 14-Gene Panel had sensitivity of 79.07% (95% CI 91.23–66.91%), specificity of 82.93% (95% CI 94.44–71.41%), PPV of 82.93% (95% CI 94.44–71.41%), and NPV of 79.07% (95% CI 91.23–66.91%) (Table 5). As a comparison, the diagnostic performance of serum PSA to distinguish Gleason score 7 subgroups was also assessed. The result showed low AUC of 0.771 (95% CI 0.871–0.671) for PSA (Figure 3H), sensitivity of 41.86% (95% CI 56.61–27.11%), specificity of 97.56% (95% CI 102.28–92.84%), PPV of 94.74% (95% CI 104.78–84.70%), and NPV of 61.54% (95% CI 73.37–49.71%) (Table 5). The result showed that the 14-Gene Panel is more accurate at distinguishing the two Gleason score 7 subgroups than PSA.

**TABLE 5:** Sensitivity, specificity, PPV, and NPV of 4-Gene-Panel and PSA to distinguish the two subgroups of Gleason score 7 patients in a prospective PCa urine cohort.

	**14-gene panel**			**PSA**		
	**Positive**	**Negative**	**Total**	**Positive**	**Negative**	**Total**
Gleason score 4 + 3 = 7	34	9	43	18	25	43
Gleason score 3 + 4 = 7	7	34	41	1	40	41
Total	41	43	84	19	65	84
Sensitivity (95% CI)	79.07 (91.23–66.91)%			41.86 (56.61–27.11)%		
Specificity (95% CI)	82.93 (94.44–71.41)%			97.56 (102.28–92.84)%		
PPV (95% CI)	82.93 (94.44–71.41)%			94.74 (104.78–84.70)%		
NPV (95% CI)	79.07 (91.23–66.91)%			61.54 (73.37–49.71)%		

### Validation of the 14-Gene Panel in a Retrospective PCa Urine Cohort

To further validate the 14-Gene Panel urine test, its diagnostic performance was assessed in another independent retrospective patient cohort. The cohort consisted of 97 patients with clinical characteristics shown in Table 1. This cohort included 47 (48.45%) higher-risk PCa and 50 (51.55%) lower-risk PCa. The urine samples collected from patients have been stored at −80°C for 5–10 years before subjected to RNA purification and qRT-PCR analysis. Similar to the prospective patient cohort, the urine collection was conducted prior to biopsy or prostatectomy. The AUC of the 14-Gene Panel for distinguishing higher- and lower-risk PCa in the retrospective cohort was 0.899 (95% CI 0.964–0.834) (*p* < 0.0001) (Figure 3I), which was very similar to that of the 14-Gene Panel in the prospective cohort (AUC of 0.897). As shown in Table 6, the 14-Gene Panel had sensitivity of 76.60% (95% CI 88.70–64.49%), specificity of 86.00% (95% CI 95.62–76.38%), PPV of 83.72% (95% CI 94.76–72.69%), and NPV of 79.63% (95% CI 90.37–68.89%). In contrast, Gleason score was found to be less accurate at distinguishing higher- and lower-risk PCa in the retrospective cohort with AUC of 0.776 (95% CI 0.854–0.698) (Figure 3J). The AUCs of the 14-Gene Panel in both retrospective and prospective cohorts were similar, showing that the 14-Gene Panel had comparable diagnostic performances in both cohorts despite the difference between the two studies.

**TABLE 6:** Sensitivity, specificity, PPV, and NPV of 4-Gene-Panel and Gleason score for diagnosis of higher-risk and lower-risk PCa in a retrospective Pca urine cohort.

	**14-gene panel**			**Gleason score**		
	**Positive**	**Negative**	**Total**	**Positive**	**Negative**	**Total**
Higher risk	36	11	47	9	36	45
Lower risk	7	43	50	0	44	44
Total	43	54	97	9	80	89
Sensitivity (95% CI)	76.60 (88.70–64.49)%			20.00 (31.69–8.31)%		
Specificity (95% CI)	86.00 (95.62–76.38)%			100 (100–100)%		
PPV (95% CI)	83.72 (94.76–72.69)%			100 (100–100)%		
NPV (95% CI)	79.63 (90.37–68.89)%			55.00 (65.90–44.10)%		

### Assessment of the 14-Gene Panel to Distinguish PCa and Benign Prostate

**FIGURE 4:**
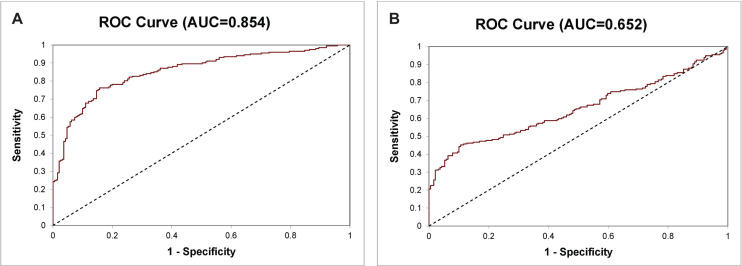
ROC curve of the 14-Gene Panel and PSA for diagnosis of PCa and benign prostate in the prospective urine cohort. ROC curve of the 14-Gene Panel **(A)** and PSA **(B)**.

There is an unmet medical need for developing diagnostic tests that can accurately distinguish PCa from benign prostate for cancer diagnosis. Besides using the 14-Gene Panel for PCa risk stratification, we tested its ability for diagnosing PCa and benign prostate in a prospective urine cohort consisting of patients with benign prostatic hyperplasia, prostatitis, and PCa (*n* = 393). A diagnostic algorithm was developed to classify samples as PCa or benign prostate using Ct_*S*_ values of the 14-Gene Panel in urinary sediment RNA. The diagnostic performance of the 14-Gene Panel was tested by comparing the diagnosis of each sample using the algorithm with pathological diagnosis. ROC analysis was performed, and the diagnostic accuracy was shown by an AUC of 0.854 (95% CI 0.892–0.816) (*p* < 0.0001) (Figure 4A). In contrast, serum PSA had an AUC of 0.652 (95% CI 0.706–0.598) (Figure 4B). This suggests that the 14-Gene Panel is more accurate than PSA for distinguishing PCa from benign prostate and has potential to become a non-invasive urine test for PCa diagnosis.

## Discussion

In this study, we identified and developed a novel biomarker panel model for an important clinical diagnostic application in the field of PCa. We showed that a 14 urinary biomarker panel can be used to distinguish higher-risk and lower-risk cancer for PCa risk stratification during treatment decision making. We showed that the 14-Gene Panel, which was initially identified and tested in cancer tissues can be used as a non-invasive test using a small volume of urine samples from patients before undergoing biopsy or surgery. We have performed bioinformatics in combination with molecular biological analysis to develop a 14-Gene Panel algorithm to identify higher-risk patients needing immediate treatment and lower-risk patients for cancer surveillance. We have shown that the 14-Gene Panel urine test had high diagnostic accuracy, especially in a subgroup of patients with Gleason scores 6 and 7 who need accurate stratification the most. We also showed that the 14-Gene Panel urine test was able to distinguish between two Gleason score 7 subgroups. In addition, we showed that the 14-Gene Panel urine test was able to distinguish PCa from benign prostate to aid PCa diagnosis.

We tested the diagnostic performance of the 14-Gene Panel in two independent prospective and retrospective studies using a convenient and non-invasive testing method with patient urine samples. The results showed that the 14-Gene Panel was capable of distinguishing higher-risk from lower-risk PCa with good accuracy in both urine sample cohorts. When we tested differential gene expression of the 14 genes in the panel in higher- and lower-risk patients, 9 genes were found to have statistically significant gene expression difference in the two risk groups. Five genes had large yet statistically insignificant difference of gene expression in the two risk groups; their contribution to the diagnostic performance of the 14-Gene Panel was assessed in the prospective and retrospective PCa urine cohorts. We found that subtracting all five genes or any one of the five genes from the 14-Gene Panel would lower the diagnostic accuracy of the panel, such as lowered sensitivity, specificity, and AUC. This suggests that each gene in the panel is important, and all genes contribute to the 14-Gene Panel.

Although the two cohorts had difference in study design (prospective study vs. retrospective study), patient ethnical background (Asian patients in the prospective cohort vs. Caucasian patients in the retrospective cohort), sample storage time (fresh sample or stored for a few months in the prospective cohort vs. stored for 5–10 years in the retrospective cohort), urine volume (15–45 ml in the prospective cohort vs. 15 ml in the retrospective cohort), and sample size (202 in the prospective cohort vs. 97 in the retrospective cohort), the diagnostic performance of the 14-Gene Panel was similar in the two cohorts with almost identical AUC value. The results of these studies suggest that it can be an accurate test for stratification of PCa patients.

Well-known prostate cancer-associated genes such as *PCA3*, *TMPRSS2*, and *CST3* were found to be upregulated in urinary sediment RNA from the higher-risk cancer group compared to those lower-risk group (Groskopf et al., 2006; Yu et al., 2010; Ito et al., 2018). *TMPRSS2* is a target gene of AR and its fusion with *ERG* occurs in 40–80% of PCa. Among the biomarkers in the 14-Gene Panel used, *VEGFA*, *PTEN*, *PIP5K1A*, *CDK1*, and *CCND1* were found to have significant differential gene expression in urinary samples between the lower- and higher-risk PCa patients. *VEGFA*, *PTEN*, *PIP5K1A*, *CDK1*, and *CCND1* in the panel are key factors involved in the PI3K/AKT pathways important for cancer survival and metastasis. Importantly, PIP5K1A/PI3K/AKT survival pathways are in part related to AR in PCa progression (Koryakina et al., 2015; Casimiro et al., 2016). Thus, changes in these genes in urine samples may be related to the development of the invasive stage of the cancer.

In order to use the active surveillance strategy to manage lower-risk PCa patients, it is important that cancer progression be monitored periodically so that the development of higher-risk PCa in the lower-risk patients can be promptly discovered, and suitable treatments can be given to the patients. Although higher-risk PCa can be diagnosed with Gleason scores of the prostate specimen in combination with PSA, cancer stage, and biopsy core with cancer based on the NCCN guidelines, it is not amenable to perform biopsy periodically to obtain Gleason score, cancer stage, and biopsy core information. Therefore, a non-invasive and convenient method such as using urine or blood specimens to perform diagnostic test should be used to monitor cancer progression in clinical practice. The fact that prostate epithelial cells are released in the urine from the prostate gland in PCa patients and urine biomarkers can be used as PCa diagnostic tests (such as PCA3) (Groskopf et al., 2006) supports the usage of the 14-Gene Panel in a urine test to distinguish between higher-risk PCa and lower-risk PCa. Thus, this urine-based non-invasive test is more advantageous than Gleason score and other prostate specimen-based measurements for PCa risk stratification and cancer progression monitoring, especially for active surveillance management.

Although some of the 14 genes in the panel have been studied before as PCa prognostic biomarkers, our combination of these genes in a panel is novel. To date, several combinations of biomarkers have been developed and studied to assess the risk of PCa using urine samples. However, those tests have limited accuracy (Jamaspishvili et al., 2010; Wu C.-L. et al., 2013; Klein et al., 2014; Tosoian et al., 2017). The 14-Gene Panel has a better combination of sensitivity, specificity, and AUC value and may potentially represent an accurate and non-invasive urine test. In the future, more clinical studies with larger patient cohorts will be conducted to confirm the accuracy of the 14-Gene Panel.

In summary, we have developed a non-invasive and convenient test using a novel 14-Gene Panel in urine samples to distinguish higher-risk from lower-risk PCa with good accuracy. This test can potentially be used for PCa risk stratification and active surveillance management in clinical practice.

## Data Availability Statement

The raw data supporting the conclusions of this article will be made available by the authors, without undue reservation, to any qualified researcher.

## Ethics Statement

The studies involving human participants were reviewed and approved by Institutional Review Board of Shenzhen People’s Hospital, Shenzhen, China (Study Number: P2014-006) and Institutional Review Board of San Francisco General Hospital, San Francisco, United States (IRB #: 15-15816). The patients provided their written informed consent prior to joining this study.

## Author Contributions

JG, DL, HJ, CZ, and KX conceptualized the study. XF, CZ, HZ, and JP contributed to the methodology. XZ and JP took part in the investigation. JG, DL, AW, and LC were in charge of the resources. HJ, JP, and JF performed the formal analysis. HJ and CZ wrote the original draft. JF and KX wrote, reviewed, and edited the manuscript. All authors contributed to the article and approved the submitted version.

## Conflict of Interest

HJ declares financial interest and employment with Olympia Diagnostics, Inc. The remaining authors declare that the research was conducted in the absence of any commercial or financial relationships that could be construed as a potential conflict of interest.

## Footnotes

1 https://seer.cancer.gov/statfacts/html/prost.html
