# Supplementary material for: Establishing a Urine-Based Biomarker Assay for Prostate Cancer Risk Stratification
Source: Front Cell Dev Biol. 2020 Dec 10;8:597961. doi: 10.3389/fcell.2020.597961 (PMC7758396; doi:10.3389/fcell.2020.597961)

Supplementary Material

Supplementary Figure 1. Box plots of genes with statistically insignificant differential gene expression in the prospective PCa urine cohort (n=202). Box plot of expression of *CDK1* (A), *EZH2* (B), *PCA3* (C), *ANXA3* (D), and *GSTP1* (E). Normalized mRNA level –Cts is defined as –Ct(sample)/Ct(actin) * 1000.

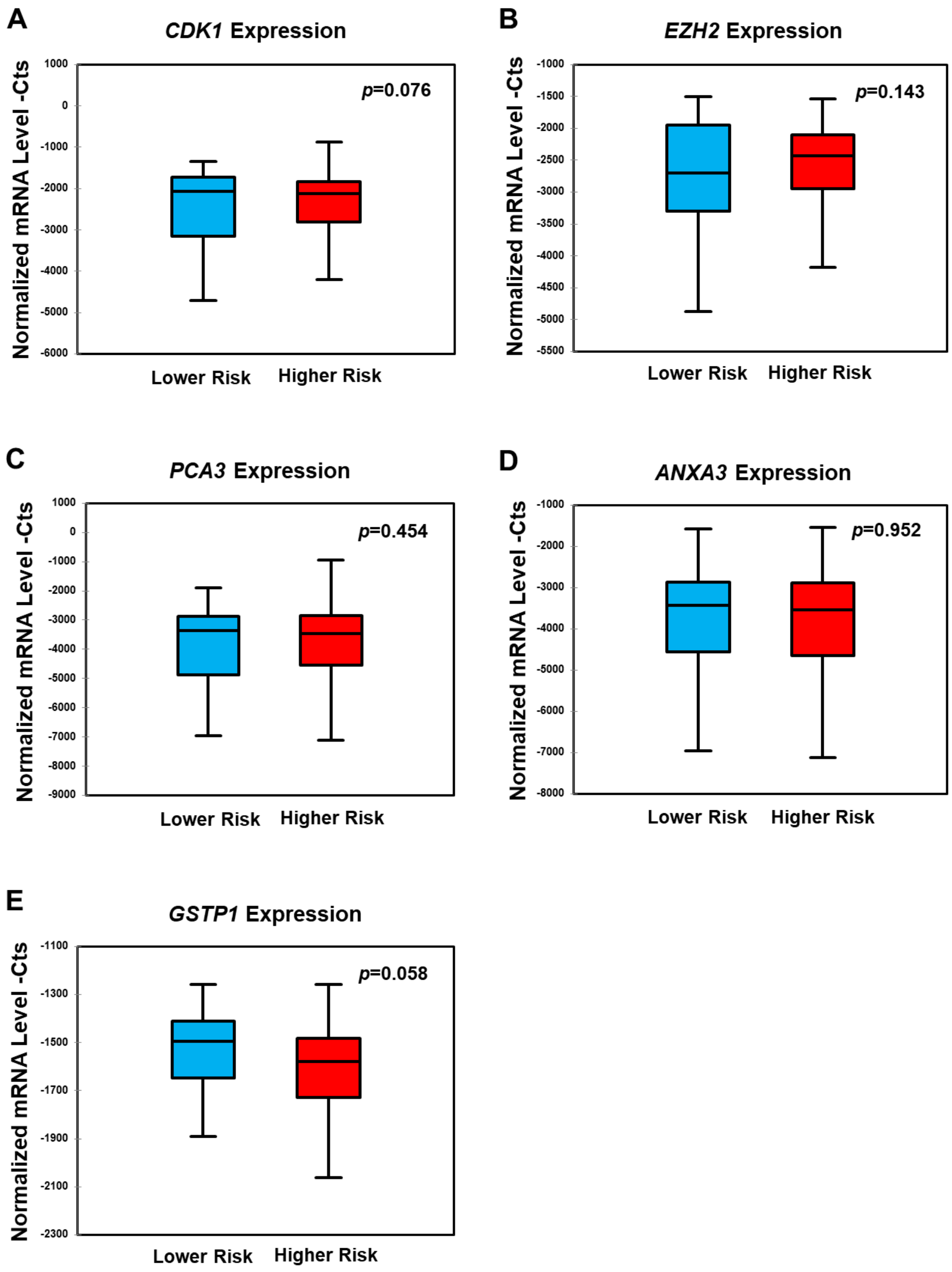

Supplement: Supplementary file 1 [file Data_Sheet_1.pdf]
